# Maintaining stroke care in Europe during the COVID-19 pandemic: Results from an international survey of stroke professionals and practice recommendations from the European Stroke Organisation

**DOI:** 10.1177/2396987320933746

**Published:** 2020-06-10

**Authors:** Diana Aguiar de Sousa, H Bart van der Worp, Valeria Caso, Charlotte Cordonnier, Daniel Strbian, George Ntaios, Peter D Schellinger, Else Charlotte Sandset, for the European Stroke Organisation

**Affiliations:** 1 Department of Neurosciences and Mental Health (Neurology), Hospital de Santa Maria/CHLN, University of Lisbon, Lisbon, Portugal; 2 Department of Neurology and Neurosurgery, Brain Center, University Medical Center Utrecht, Utrecht, the Netherlands; 3 Stroke Unit and Division of Internal and Cardiovascular Medicine, University of Perugia, Perugia, Italy; 4 U1172 - LilNCog - Lille Neuroscience & Cognition, CHU Lille, Univ. Lille, Inserm, Lille, France; 5 Department of Neurology and Neurosciences, Helsinki University Hospital and Helsinki University, Helsinki, Finland; 6 Department of Internal Medicine, Faculty of Medicine, School of Health Sciences, University of Thessaly, Larissa, Greece; 7 John Wesling Medical Center Minden, UK RUB, Minden, Germany; 8 Stroke Unit, Department of Neurology, Oslo University Hospital, Oslo, Norway

**Keywords:** Stroke, health services, COVID-19, stroke care

## Abstract

**Introduction:**

The coronavirus disease 2019 (COVID-19) pandemic has been placing an overwhelming burden on health systems, thus threatening their ability to operate effectively for acute conditions in which treatments are highly time sensitive, such as cerebrovascular disorders and myocardial infarction. As part of an effort to reduce the consequences of this outbreak on health service delivery to stroke patients, the European Stroke Organisation has undertaken a survey aimed at collecting information on the provision of stroke care during the pandemic.

**Methods:**

Cross-sectional, web-based survey, conducted from 26 March through 1 April 2020 among stroke care providers, focused on reorganisation of health services, the delivery of acute and post-acute stroke care and the availability of personal protective equipment.

**Results:**

A total of 426 stroke care providers from 55 countries completed the survey, most of whom worked in Europe (n = 375, 88%) and were stroke physicians/neurologists (n = 334, 78%). Among European respondents, 289 (77%) reported that not all stroke patients were receiving the usual care in their centres and 266 (71%) estimated that functional outcomes and recurrence rates of stroke patients would be negatively affected by the organisational changes caused by the pandemic. The areas considered as being most affected were acute care and rehabilitation. Most professionals had to adapt their activities and schedules and more than half reported shortage of protective equipment.

**Discussion:**

Strategies to maintain availability of stroke care during the COVID-19 outbreak are crucial to prevent indirect mortality and disability due to suboptimal care.

**Conclusion:**

European Stroke Organisation proposes a set of targeted actions for decision makers facing this exceptional situation.

## Background and objectives

The coronavirus disease 2019 (COVID-19)^1^ outbreak has been spreading rapidly around the world and places an overwhelming burden on emergency systems, health-care facilities and health-care workers. Also, governmental instructions together with the response pattern of the population, due to fear of infection in medical facilities, have led to a situation where several elective consultations and procedures have been postponed. Resource restrictions for urgent health conditions, such as stroke and myocardial infarction, may have a significant impact on mortality and morbidity, potentially even larger than that of COVID-19 disease itself. An increase in mortality associated with treatable conditions has been demonstrated during previous viral outbreaks such as the 2009 influenza A H1N1 virus, in which a greater surge in hospital admissions was associated with significant increases in the mortality attributable to other diseases, including stroke and acute myocardial infarction.^2,3^

Currently, data are not available regarding the impact of the pandemic on access to and delivery of stroke care in Europe. As part of the ongoing effort to reduce the consequences of COVID-19 on health service delivery to stroke patients, the European Stroke Organisation (ESO) has undertaken a survey aimed at collecting information on the current provision of stroke care. These results should provide relevant information to direct activities aimed at promoting the best possible care to all stroke patients during the pandemic and support stroke physicians and other professionals working in the field.

## Methodology

### Study design and participants

A cross-sectional, web-based survey for stroke care providers was administered from 26 March through 1 April 2020. As of 31 March 2020, 423,946 COVID-19 cases and 26,694 COVID-19-related deaths had been reported in the European region, with 11,591 deaths reported from Italy alone and a total number of infected people in Spain already exceeding those reported in China.^4^

A 12-item draft survey instrument comprising 10 closed-ended questions and two open-ended questions was developed by the first and senior authors and circulated and tested among the members of the executive committee of ESO. Refinements were made as required to facilitate better comprehension and to organise the questions. The provider used for survey application and server capacity was Lamapoll (http://lamapoll.de). The final survey was made accessible through a link that was distributed by email to all ESO members and advertised in the official social media channels of the society. ESO is pan-European scientific non-profit organisation of stroke researchers and physicians, national and regional stroke societies and lay organisations that was founded in 2007 and had 2099 members at the time of this survey. Before starting, participants were informed of the purpose of the study, target respondents and confidentiality. Participants had to confirm they wished to submit their final responses at the end and a message acknowledging successful completion of the questionnaire was sent by the server. Cookies and IP address analysis were used to identify potential duplicate entries from the same user, which were avoided by preventing users to access the survey twice. Total completion time was recorded. The survey was anonymous and confidentiality of information was assured. Participation in this survey was voluntary and was not compensated. The data that support the findings of this study are available from the corresponding author upon reasonable request.

### Outcomes

We focused on the reorganisation of health services and reallocation of professionals, delivery of acute and post-acute stroke care and availability of personal protective equipment. Open-ended text fields for comments and suggestions were also included. A copy of the questionnaire is provided in full in the Data Supplement.

Demographic data were self-reported by the participants, including occupation, type of hospital and geographical location (country). The World Health Organisation definition of the European region was applied. Responses from stroke care providers working outside of the European regions were excluded from the primary analysis but provided as Supplementary Material. Because Italy and Spain were the most affected countries in Europe at the time of the survey, sensitivity analyses excluding respondents from other countries were performed for items related with delivery of stroke care and availability of personal protective equipment.

### Statistical analysis

Data are presented as numbers and percentages. Data analysis was performed using Microsoft Excel for Mac 2011.

## Results

### Participants

The survey was completed by 426 participants from 55 countries on six continents. No responses were excluded. Table 1 shows the demographic and occupational characteristics of the European participants. The distribution per country is described in Supplementary Table 1. Most of the respondents were stroke physicians/neurologists (n = 334, 78%) from Europe (n = 375, 88%) and working at tertiary hospitals (n = 321, 75%). The remaining participants were interventionalists (5%), rehabilitation physicians (3%), allied health-care professionals (9%) and other professionals working in the field (resident physicians, emergency physicians and intensivists; 4%). Among European respondents, 303 (81%) were stroke physicians/neurologists (Table 1) and 111 (30%) reported having treated patients with stroke and COVID-19. A summary of the responses from the 51 participants working outside Europe is provided in the Data Supplement.

**Table 1.** Demographic and occupational characteristics of European respondents.

	No. (%)
Occupation	375 (88)
Stroke physician/neurologist	303 (81)
Interventionalist	18 (5)
Allied health-care professional	30 (8)
Rehabilitation physician	15 (4)
Other	9 (2)
Type of hospital	375 (88)
Tertiary care centre	279 (74)
Community hospital	96 (26)
Current working situation	375 (88)
Similar schedule and activities	128 (34)
Similar schedule but doing new tasks outside of stroke care	55 (15)
Extended working hours	62 (17)
Mostly working from home	68 (18)
Other	62 (17)

### Delivery of stroke care

**Figure 1.**
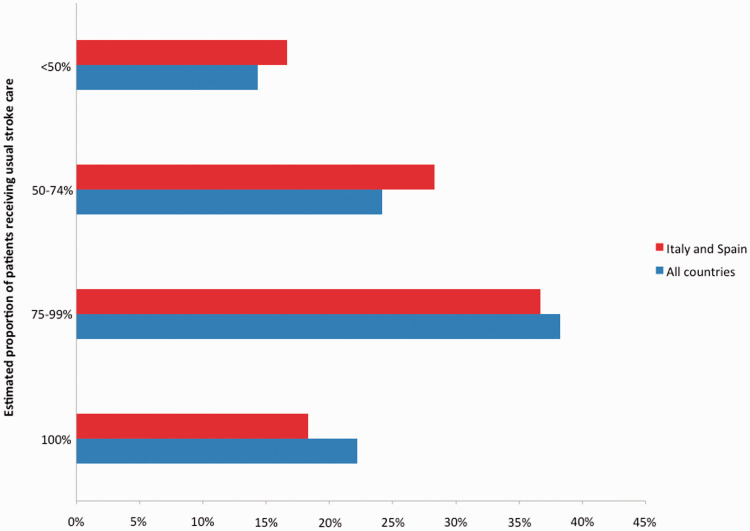
Estimated proportion of patients receiving usual stroke care in the respondents’ stroke centre.

Among stroke care providers working in the Europe, 289 (77%) reported that not all stroke patients were receiving the usual care in their centres, with 141 (38%) estimating that this was happening in more than one quarter of patients (Table 2). Of the 60 respondents from Italy and Spain, 49 (82%) reported being unable to provide the usual care to all stroke patients (Figure 1).

**Table 2.** Delivery of stroke care according to respondents from the European region.

	Total (n = 375)	Community hospital (n = 96)	Tertiary care centre (n = 279)
The estimated proportion of patients receiving usual stroke care in my centre is 100%	80 (21)	22 (23)	58 (21)
The estimated proportion of patients receiving usual stroke care in my centre is 75% to 99%	146 (39)	35 (37)	111 (40)
The estimated proportion of patients receiving usual stroke care in my centre is 50% to 74%	98 (26)	25 (26)	73 (26)
The estimated proportion of patients receiving usual stroke care in my centre is <50%	45 (12)	13 (14)	32 (12)
The stroke code pathways have been affected^a^	77 (25)	12 (17)	65 (28)
Stroke patients are now directed to other hospitals^a^	12 (4)	4 (6)	8 (3)
There is a lack of beds for stroke patients^a^	35 (12)	4 (6)	31 (13)
Endovascular treatment is currently not performed if there is a need for intensive care^b^	10 (3)	2 (3)	8 (3)
We did endovascular treatment before, but now we are not able to provide it in our centre^b^	7 (2)	1 (1)	6 (2)
We avoid admitting patients whenever possible^a^	65 (21)	13 (19)	52 (22)
Several important ancillary exams are not available now^a^	68 (22)	12 (17)	56 (24)
The TIA clinic has been closed^a^	31 (10)	5 (7)	26 (11)

Note: Values are given as n (%). TIA: Transient Ischemic attack.

^a^Answers from stroke physicians and/or neurologists working in Europe (total n = 303); tertiary centres n = 234; community hospitals n = 69).

^b^Answers from stroke physicians and/or neurologists or interventionalists working in Europe (total: n = 321); tertiary centres n = 246; community hospitals n = 75).

Two hundred sixty-six European participants (71%) estimated that functional outcomes and recurrence rates of stroke patients would be affected by the changes in stroke care related to the COVID-19 outbreak. The areas of stroke care considered as being the most affected by the current situation were rehabilitation (n = 179, 48%) and acute stroke care (n = 125, 33%).

For topics related to medical management of stroke patients, only responses from stroke physicians or neurologists (n = 334) were included (Table 2); 77 (25%) European participants reported that stroke code pathways were affected at their centres. Also, 65 (21%) reported that their centre avoided admitting patients whenever possible and 35 (12%) described lack of beds for stroke patients, while 12 (4%) had been forced to direct stroke patients to other hospitals. About one in five physicians reported that several basic ancillary examinations were no longer available (n = 68, 22%). Closure of transient ischemic attack clinics was described by 31 European respondents (10%).

For questions related to endovascular treatment, we considered responses provided by stroke physicians/neurologists and interventionalists. In Europe, 17 (5%) reported problems in endovascular treatment, particularly that this was not possible when there was a need for intensive care (n = 10, 3%) or that they were no longer able to provide this treatment at their centre at all (n = 7, 2%).

The most common comment included in the open-ended text fields was the perception of a clear drop in hospital admissions for stroke and later arrival during this period.

### Activity and workload of stroke care providers

About two-thirds of European participants reported changes in their working situation, either related to new activities or modifications in the work schedules (Table 2). Sixty-two (17%) described extended working hours due to a lack of personnel and 55 (15%) reported the need to contribute to new tasks outside stroke care. Almost one of every five professionals was doing most of the work from home. Compared with those working in tertiary care centres, participants working in community hospitals were more likely to have similar schedules and activities as before the outbreak and less likely to work from home (Supplementary Figure 2). Several specific strategies for team management were reported, including organisation of separate teams for different activities and rotation schemes that include periods of isolation.

### Protective equipment

Shortage of personal protective equipment was reported by more than half of the European respondents (n = 204, 54%) and by 65% of the stroke care providers from Italy and Spain.

## Discussion

This cross-sectional survey including 375 stroke care providers from Europe revealed profound changes in delivery of stroke care early during the early phase of the COVID-19 pandemic. These answers reflect the participants’ experiences and perspectives at a specific point in time during the outbreak in which the infection was rapidly spreading.

Only 21% of respondents considered that, in their centres, all stroke patients were still receiving the usual acute and post-acute care, and more than 70% estimated that functional outcomes and recurrence rates of stroke patients would be affected by the changes in stroke care related with the outbreak. European stroke care providers reported their most significant challenges centred on acute care and rehabilitation. Although most professionals adapted their activities and schedules, substantial challenges were noted in maintaining the pathways for acute stroke patients and the quality of inpatient care, particularly in having sufficient resources to guarantee availability of beds for all patients and proper etiologic investigation.

Acute reperfusion strategies, secondary stroke prevention and rehabilitation, among several other interventions, have the potential to provide a significant and long-lasting benefit to patients with stroke, and the targets for the implementation of these treatments have been set before.^5–8^ Even in a setting of increasingly limited resources, efforts to preserve essential services and to provide the maximum benefit for the population have to be made.^9^ Importantly, rehabilitation should be also included in decisions regarding which health services are essential, as delayed access to these therapies can compromise health and functional outcomes.^10^ To overcome these demands, local and national health managers need to carefully plan the extent to which acute and post-acute stroke services should operate during the pandemic peaks and how their continuity can be maximised, mitigating the risk of a collapse of stroke care. Also, multidisciplinary collaboration should be maintained to ensure a smooth workflow, as is the case with anaesthesia and intensive care for patients receiving endovascular treatment for ischaemic stroke.

One in five respondents had treated patients with stroke and COVID-19. The current evidence suggests that COVID-19 often triggers a strong inflammatory reaction that may predispose to ischaemia and thrombosis.^11^ As this combination of diseases is likely to become more frequent, it is important to ensure that effective reperfusion therapies and stroke unit care are also available for these patients, in parallel with a safe and efficient medical environment. Also, proper evaluation should not be delayed, regardless of concerns about possible infection, and confirmed patients with COVID-19 should be transferred to the designated medical institutions for further optimal medical treatment when indicated.

Shortage of personal protective equipment was a common concern, as it was reported by more than half of the respondents. Stroke teams are in the frontline of the pandemic, meeting patients in the acute setting with unknown COVID-19 status. Establishing protected stroke code pathways^12,13^ will contribute to adequate acute management of stroke patients with confirmed infection or unknown COVID-19 status.

Furthermore, as highlighted by several respondents, it is crucial to communicate with the community so that people know they can continue to safely seek appropriate care when stroke symptoms ensue, and that this is critical.

This report was not meant to be all inclusive of the undergoing changes and difficulties faced by health-care providers, hospitals and other health-care systems to maintain delivery of stroke care during this outbreak but to raise awareness among decision makers of the importance of preserving capacity to provide appropriate care to stroke patients during the COVID-19 pandemic.

After consideration of these key issues, ESO reinforces the following recommendations:

- Stroke care is an essential health service and should be prioritised in the strategic planning to manage the demands related to the response to the COVID-19 outbreak.
- The general population should be informed that stroke is an emergency and treatment is still available, so they must continue calling emergency services immediately in case of suspected stroke. Public education campaigns can be an effective way of raising awareness.
- Acute stroke teams are frontline workers. Patients with unknown COVID-19 status should be evaluated under ‘protected stroke code’^12^ and therefore access to appropriate personal protective equipment is mandatory for all team members as well as clear protocols for individual protection.
- Stroke registers and researchers must deploy resources to evaluate the effects of the COVID-19 pandemic on case volumes, time metrics and clinical outcomes. We suggest that the available data sources are used to assess changes in number hospital admissions, baseline characteristics, in-hospital workflow metrics, treatment rates and functional outcomes of stroke patients during this period.
- As the outbreak progresses over time, countries should be able to increasingly resume post-acute care services for stroke patients, such as rehabilitation. As the current situation is likely to influence the organisation of health care for the next months or years, this may require the adaptation and expansion of rehabilitation facilities and staff to meet the communities’ needs, both now and in the longer term. Moreover, strategies to develop and implement telehealth services should be promoted and supported by health authorities.

## Limitations

This study has several limitations. First, most participants were from tertiary centres and countries are not equally represented, limiting the scope of the conclusions and the generalisation of these findings. Second, the survey was carried out during a short period and lacked longitudinal follow-up. Because of the increasing dissemination of the infection, health-care strain may worsen and the long-term effects remain unknown. Third, we were unable to distinguish whether the respondents worked in the same hospital or region or in different regions. Finally, response bias may exist, and non-respondents may either be more likely to work in the most affected regions and not have the time to participate, or work in the least affected regions.

## Conclusions

Strain of health services during the COVID-19 pandemic has been causing major disruptions to stroke care in Europe, with likely serious and long-term implications. Shortage of personal protection equipment has been common among stroke care providers. Efforts to maintain stroke teams and safe provision of stroke care, including reperfusion treatments, should be prioritised.

## Supplemental Material

sj-pdf-1-eso-10.1177_2396987320933746 - Supplemental material for Maintaining stroke care in Europe during the COVID-19 pandemic: Results from an international survey of stroke professionals and practice recommendations from the European Stroke Organisation Click here for additional data file. Supplemental material, sj-pdf-1-eso-10.1177_2396987320933746 for Maintaining stroke care in Europe during the COVID-19 pandemic: Results from an international survey of stroke professionals and practice recommendations from the European Stroke Organisation by Diana Aguiar de Sousa, H Bart van der Worp, Valeria Caso, Charlotte Cordonnier, Daniel Strbian, George Ntaios, Peter D Schellinger, Else Charlotte Sandset and for the European Stroke Organisation in European Stroke Journal
