# Supplementary material for: Maintaining stroke care in Europe during the COVID-19 pandemic: Results from an international survey of stroke professionals and practice recommendations from the European Stroke Organisation
Source: Eur Stroke J. 2020 Jun 10;5(3):230–6. doi: 10.1177/2396987320933746 (PMC7538757; doi:10.1177/2396987320933746)

**DATA SUPPLEMENT**

**Supplementary methods**

Questionnaire

EUROPEAN STROKE ORGANISATION

## Disruptions in Stroke Care during COVID-19 pandemic

Dear colleagues and friends,

The focus of many healthcare workers and hospital administrators is now rightly on managing the COVID-19 pandemic. It is however imperative for our stroke patients that we keep our services throughout the pandemic. Perhaps not in the same form as under normal conditions, but we must strive to ensure good acute stroke pathways and to offer the best treatment possible under these challenging conditions. We must look for solutions, be creative and take care of both patients and colleagues.

In order to get an overview of the current situation across Europe we kindly invite you to participate in a short survey preferably by this 1 April.

The results of the survey may be used to write a statement on behalf of ESO. Data will be assessed and analysed on a country-level basis, without access to personal data.

We thank Diana Aguiar de Sousa for contributing to this survey.

Best wishes,

On behalf of the ESO Executive Committee,

Bart van der Worp | ESO President

Else Charlotte Sandset | ESO Secretary General

### 1. What is your current working situation?

- ◯ Similar schedule and activities at the hospital
- ◯ Mostly working from home (telephone consultation, other activities)
- ◯ Same activities as before, but extended schedule, due to lack of personnel
- ◯ Similar schedule at the hospital, but doing different tasks, outside of stroke care
- ◯ Extended working hours, mostly outside of stroke care
- ◯ Other, please specify

**2. Has stroke care been affected at your hospital?** **(Please tick all that apply)**

☐ No, we are able to provide the same care as before

☐ We are able to provide the same care but with limited manpower/resources

☐ The stroke code pathways have been affected

☐ Stroke patients are now directed to other hospitals

☐ Endovascular treatment is currently not performed if there is a need for intensive care

☐ We did endovascular treatment before, but now we are not able to provide it in our centre

☐ There is a lack of beds for stroke patients

☐ We avoid admitting patients whenever possible by organising ancillary investigations outside the hospital or with outpatient visits

☐ Several important exams (e.g. Holter monitoring, ultrasound, echocardiography) are not available right now

☐ Our TIA clinic has been closed

☐ Other, please specify

**3. Regarding personal protective equipment for personnel involved in stroke care**

○ There is a shortage of protective equipment

○ We have been using appropriate protective equipment, according to the available guidelines

**4. Can you provide an estimate of how many stroke patients are still receiving the usual stroke care in your centre (acute and post-acute care services)?**

☐ 100%

☐ 75-99%

☐ 50-74%

☐ <50%

☐ Comment

**5. Do you believe that functional outcomes and recurrence rates of stroke patients will be affected by these changes in health care?**

- Yes
- No
- I am not able to answer

**6. Which of the following areas of stroke care do you see as being affected most by the current situation and thus require particular attention (please choose one option):**

- Primary stroke prevention
- Acute stroke care
- Secondary stroke prevention
- Rehabilitation

**7. Have you seen patients with stroke and COVID-19 infection (infection before or after stroke)?**

- Yes
- Not yet

**8. Do you have any suggestion on ESO initiatives that could be useful to support stroke teams facing these challenges?**

**9. Is there a message / experience / learning you would like to share with the ESO community?**

**10. Please indicate the country where you work**

**11. At what type of hospital do you mainly work**

- University/tertiary care centre
- Community hospital

**12. Please indicate your position**

- ○ Stroke physician
- ○ Neurologist
- ○ Rehabilitation physician
- ○ Radiologist / Interventionalist
- ○ Allied health professional
- ○ Other, please specify

## Supplementary Results

### Respondents working outside Europe

The demographic and occupational characteristics of the non-European participants are described in Supplementary Table 2. Most of the 51 respondents working outside of Europe reported not being able to provide the usual care to all patients in their centres (n=39, 76%). Likewise, most estimated that functional outcomes and recurrence rates of stroke patients would be affected by the changes in stroke care related to the COVID-19 pandemic (n=37, 73%). The areas of stroke care considered as being the most affected by the current situation were rehabilitation (n=29, 57%) and acute stroke care (n=13, 26%). Similar proportions of respondents reported changes in delivery of stroke care, as described in Supplementary Table 3.

Among respondents working outside Europe, 22 (43%) maintained a similar schedule and activities as before, while the remaining participants reported changes as described in Supplementary Table 2. Shortage of personal protective equipment was reported by 33 participants (65%).

**Supplementary table 1. Distribution of respondents per country**

| | |
|---|---|
| Argentina | 3 |
| Australia | 3 |
| Austria | 7 |
| Belgium | 17 |
| Brazil | 1 |
| Bulgaria | 1 |
| Colombia | 7 |
| Costa Rica | 1 |
| Croatia | 4 |
| Czechia | 2 |
| Denmark | 4 |
| Estonia | 1 |
| Finland | 4 |
| France | 15 |
| Germany | 38 |
| Greece | 9 |
| Guatemala | 1 |
| Hungary | 1 |
| India | 14 |
| Indonesia | 1 |
| Iraq | 1 |
| Ireland | 3 |
| Israel | 8 |
| Italy | 40 |
| Japan | 1 |
| Kazakhstan | 1 |
| Kyrgyzstan | 1 |
| Lithuania | 3 |
| Luxembourg | 1 |
| Malaysia | 1 |
| Malta | 1 |
| Mexico | 9 |
| Netherlands | 15 |
| Nigeria | 1 |
| North Macedonia | 2 |
| Norway | 10 |
| Panama | 1 |
| Philippines | 1 |
| Poland | 6 |
| Portugal | 67 |
| Romania | 8 |
| Russia | 4 |
| Saudi Arabia | 1 |
| Serbia | 5 |
| Singapore | 1 |
| Slovakia | 1 |

| | |
|---|---|
| South Korea | 1 |
| Spain | 23 |
| Sweden | 5 |
| Switzerland | 21 |
| Turkey | 3 |
| Ukraine | 7 |
| United Kingdom | 37 |
| United States | 1 |
| Vietnam | 1 |

**Supplemental Table 2. Demographic and Occupational Characteristics of Non-European Respondents**

| | No. (%) |
|---|---|
| **Occupation** | 51 (12) |
| Stroke physician/neurologist | 31 (72) |
| Interventionalist | 3 (6) |
| Allied Healthcare professional | 7 (14) |
| Rehabilitation physician | 0 (0) |
| Other | 10 (20) |
| **Type of Hospital** | 51 (12) |
| Tertiary care centre | 42 (82) |
| Community Hospital | 9 (18) |
| **Current Working situation** | 51 (12) |
| Similar schedule and activities | 22 (43) |
| Similar schedule but doing new tasks outside of stroke care | 10 (20) |
| Extended working hours | 3 (6) |
| Mostly working from home | 12 (24) |
| Other | 4 (8) |

**Supplementary Table 3. Delivery of Stroke Care according to non-European respondents**

| | No. (%) |
|---|---|
| **The estimated proportion of patients receiving usual stroke care in my centre is 100%** | 12 (24) |
| **The estimated proportion of patients receiving usual stroke care in my centre is 75-99%** | 17 (33) |
| **The estimated proportion of patients receiving usual stroke care in my centre is 50-74%** | 5 (10) |
| **The estimated proportion of patients receiving usual stroke care in my centre is <50%** | 17 (33) |
| **The stroke code pathways have been affected*** | 11 (35) |
| **Stroke patients are now directed to other hospitals*** | 1 (3) |
| **There is a lack of beds for stroke patients*** | 2 (6) |
| **Endovascular treatment is currently not performed if there is a need for intensive care §** | 3 (9) |
| **We did endovascular treatment before, but now we are not able to provide it in our centre §** | 1 (3) |
| **We avoid admitting patients whenever possible*** | 4 (13) |
| **Several important ancillary exams are not available now*** | 4 (13) |
| **The TIA clinic has been closed*** | 2 (6) |

*Answers from stroke physicians and/or neurologists (n=31)
§ Answers from stroke physicians and/or neurologists or Interventionalists (n=34)

**Supplementary Figure 1. Distribution of respondents per continent**

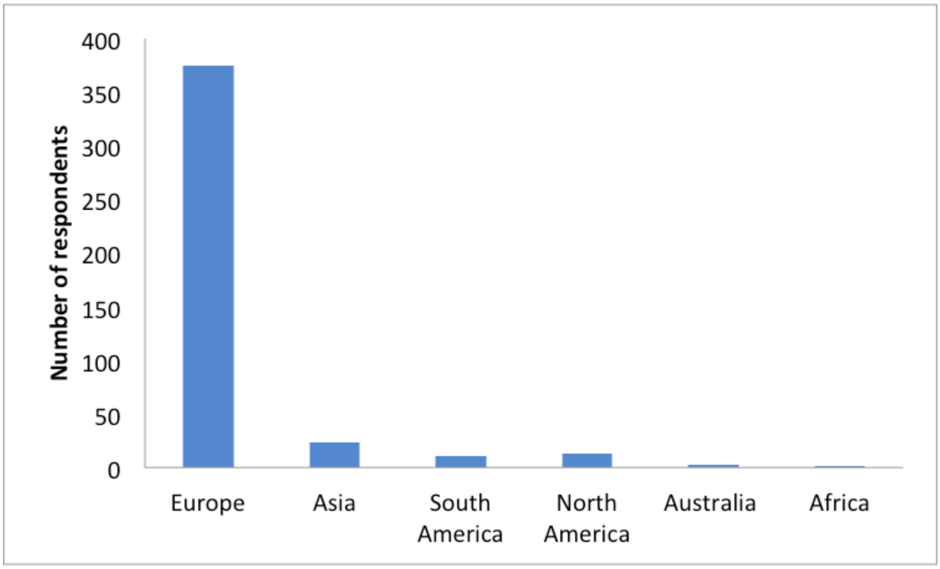

**Supplementary Figure 2. Changes in working tasks and schedules of stroke care providers**

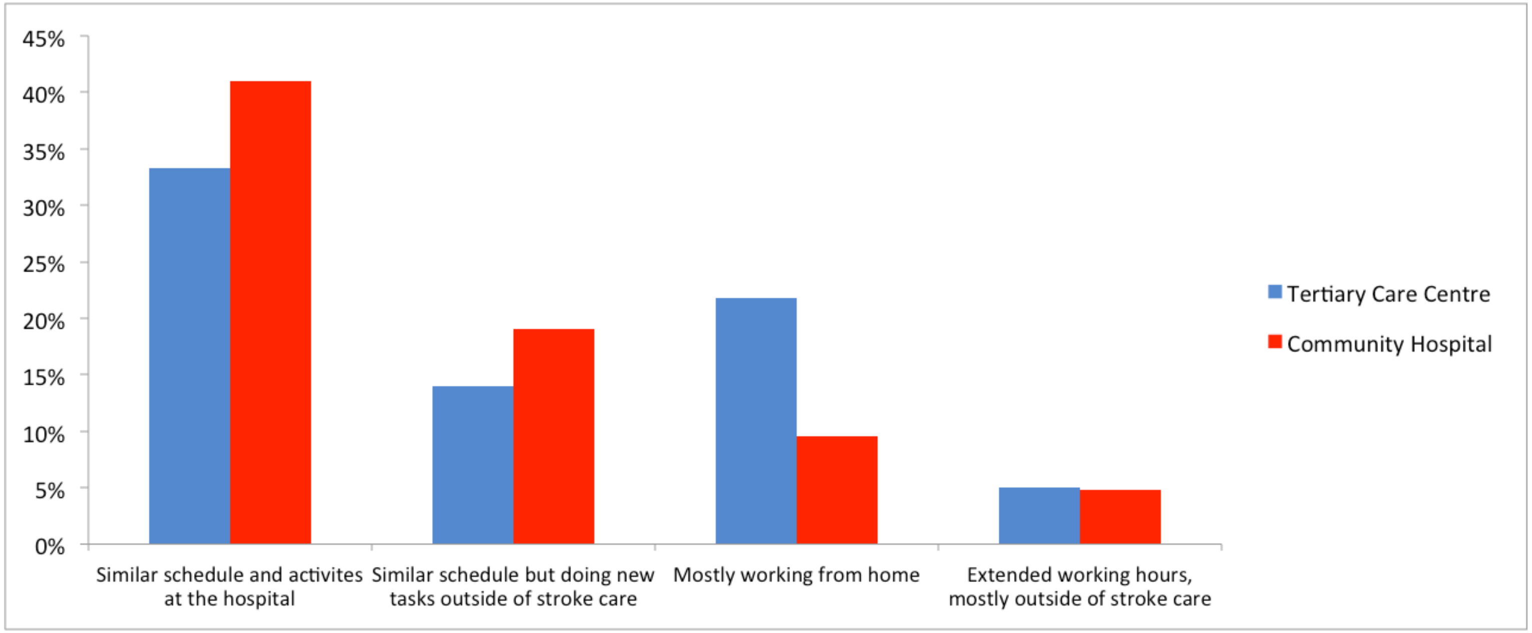

Supplement: sj-pdf-1-eso-10.1177_2396987320933746 - Supplemental material for Maintaining stroke care in Europe during the COVID-19 pandemic: Results from an international survey of stroke professionals and practice recommendations from the European Stroke Organisation [file sj-pdf-1-eso-10.1177_2396987320933746.pdf]
